# GNF-7, a novel FLT3 inhibitor, overcomes drug resistance for the treatment of FLT3‑ITD acute myeloid leukemia

**DOI:** 10.1186/s12935-023-03142-y

**Published:** 2023-11-30

**Authors:** Xinhua Xiao, Peihong Wang, Weina Zhang, Jiayi Wang, Mansi Cai, Hua Jiang, Yingli Wu, Huizhuang Shan

**Affiliations:** 1 Department of Hematology and Oncology, Guangzhou Women and Children’s Medical Center, Guangzhou Medical University, Guangzhou, 510623 China; 2 Department of Clinical Laboratory Medicine, Guangdong Provincial People’s Hospital (Guangdong Academy of Medical Sciences), Southern Medical University, Guangzhou, 510000 Guangdong China; 3 Department of Hematology, Guangzhou First People’s Hospital, South China University of Technology, Guangzhou, 510000 Guangdong China; 4 Hongqiao International Institute of Medicine, Shanghai Tongren Hospital/Faculty of Basic Medicine, Chemical Biology Division of Shanghai Universities E-Institutes, Key Laboratory of Cell Differentiation and Apoptosis of the Chinese Ministry of Education, Research Units of Stress and Tumor (2019RU043), Shanghai Jiao Tong University School of Medicine, Chinese Academy of Medical Sciences, Shanghai, 200025 China

**Keywords:** Acute myeloid leukemia, FLT3-ITD, GNF-7, Drug resistance

## Abstract

**Background:**

Acute myeloid leukemia (AML) with FMS-like tyrosine kinase 3 internal tandem duplication (FLT3-ITD) mutation accounts for a large proportion of AML patients and diagnosed with poor prognosis. Although the prognosis of FLT3-ITD AML has been greatly improved, the drug resistance frequently occurred in the treatment of FLT3 targeting drugs. GNF-7, a multitargeted kinase inhibitor, which provided a novel therapeutic strategy for overriding leukemia. In this study, we explored the antitumor activity of GNF-7 against FLT3-ITD and clinically-relevant drug resistance in FLT3 mutant AML.

**Methods:**

Growth inhibitory assays were performed in AML cell lines and Ba/F3 cells expressing various FLT3 mutants to evaluate the antitumor activity of GNF-7 in vitro. Western blotting was used to examine the inhibitory  effect of GNF-7 on FLT3 and its downstream pathways. Molecular docking and cellular thermal shift assay (CETSA) were performed to demonstrate the binding of FLT3 to GNF-7. The survival benefit of GNF-7 in vivo was assessed in mouse models of transformed Ba/F3 cells harboring FLT3-ITD and FLT3-ITD/F691L mutation. Primary patient samples and a patient-derived xenograft (PDX) model were also used to determine the efficacy of GNF-7.

**Results:**

GNF-7 inhibited the cell proliferation of Ba/F3 cells expressing FLT3-ITD and exhibited potently anti-leukemia activity on primary FLT3-ITD AML samples. Moreover, GNF-7 could bind to FLT3 protein and inhibit the downstream signaling pathway activated by FLT3 including STAT5, PI3K/AKT and MAPK/ERK. In vitro and in vivo studies showed that GNF-7 exhibited potent inhibitory activity against FLT3-ITD/F691L that confers resistant to quizartinib (AC220) or gilteritinib. Importantly, GNF-7 showed potent cytotoxic effect on leukemic stem cells, significantly extend the survival of PDX model and exhibited similar therapy effect compared with gilteritinib.

**Conclusions:**

Our results show that GNF-7 is a potent FLT3-ITD inhibitor and may become a promising lead compound applied for treating some of the clinically drug resistant patients.

**Supplementary Information:**

The online version contains supplementary material available at 10.1186/s12935-023-03142-y.

## Background

Activating mutations of FMS-like tyrosine kinase 3 (*FLT3*) account for approximately 30% of acute myeloid leukemia (AML) patients and can be either FLT3 internal tandem duplication (FLT3-ITD) mutations or FLT3 tyrosine kinase domain point mutations (FLT3-TKD) .^1^ In addition, mutation of FLT3-ITD occurs in 25% of diagnosed AML patients and which are associated with poor outcomes [1, 2]. Whereas, FLT3-TKD have not been associated with a consistent prognostic impact on AML patients [3]. FLT3-ITD lead to receptor dimerization and ligand-independent constitutive activation of downstream signal transduction pathways, such as mitogen-activated protein kinase (MAPK) signal transducer and activator of transcription 5 (STAT5), which maintain the leukemia cells proliferation [4–6]. With FLT3 inhibitors such as sorafenib, quizartinib (AC220), midostaurin, crenolanib and gilteritinib developed, AML patients gained great benefits from the previous chemotherapy [7]. However, the duration of clinical responses to FLT3 inhibitors is transient because of rapid and high rates of drug resistance [8–10].

Pan-resistant FLT3-ITD/F691L mutation is the common and stubborn resistant mechanism and showed resistant to currently in clinical used FLT3 inhibitors such as sorafenib, midostaurin, crenolanib and gilteritinib [10–12]. Thus, it is paramount to find effective compounds to overcome the drug resistance caused by FLT3-ITD/F691L.

Here, we identified a novel inhibitor, GNF-7, previously reported as a BCR::ABL1 inhibitor [13], which also shows a unique kinase inhibitory effect on FLT3 kinase and provides potent inhibition of FLT3 phosphorylation and downstream signaling pathways in FLT3-ITD expressing cell lines. Interestingly, GNF-7 selectively bind with FLT3-ITD protein. In addition, GNF-7 shows strong anti-leukemia effects against AML cells harboring FLT3-ITD and FLT3-ITD/F691L in vitro. Of note, GNF-7 exerts the same therapeutic effect as gilteritinib in a FLT3-ITD mouse xenograft model and significantly prolongs the survival of FLT3-ITD/F691L leukemia mice. In two FLT3-ITD patient-derived AML xenotransplantation models, GNF-7 also demonstrated excellent therapeutic efficacy. Our study suggests that GNF-7 may be a promising drug in the treatment of FLT3-ITD AML.

## Materials and methods

### Cell lines and compounds

MV4-11, MOLM-13, U937 and THP-1 cells were cultured with RPMI-1640 (Gibco) containing 10% FBS (Corning). Ba/F3 cells expressing FLT3-ITD, FLT3-ITD/F691L, FLT3-ITD/D835Y, FLT3-ITD/D835V, FLT3-ITD/D835F, FLT3-ITD/Y842C, BCR::ABL1/P190 (P190) and BCR::ABL1/T315I (T315I) were generated by retroviral infection as previously described^11^ and cultured with RPMI-1640 containing 10% FBS. AC220, imatinib and dasatinib were purchased from Selleck (Shanghai, China), GNF-7 was purchased from CSNpharm (Shanghai, China), gilteritinib was purchase from AbMole (Shanghai, China).

### Mononuclear cells isolated from bone marrow

Bone marrow samples were collected from three patients with diagnosed FLT3-ITD-AML (detailed information for these patients are provided in Additional file 1: Table S1). Mononuclear cells (MNCs) were then separated from umbilical cord blood or bone marrow samples as previously reported [14] and supplemented with 100 U/mL penicillin and 100 μg/mL streptomycin (Sigma Aldrich). This study was approved by the Institutional Review Board of the Guangzhou Women and Children’s Medical Center affiliated to Guangzhou Medical University and Guangzhou First People's Hospital. Informed consent for the in vitro drug testing studies was obtained in accordance with the declaration of Guangzhou Women and Children’s Medical Center affiliated to Guangzhou Medical University and Guangzhou First People's Hospital.

### Cell growth inhibition assay

Normalized cell proliferation detected in the study were carried out using the CellTiter-Glo® Luminescent Cell Viability Assay as described previously [14]. Leukemia cell lines and primary cells were seeded into 96-well cell culture plates at a density of 10^4^ and 2 × 10^4^ cells per well, and then added with indicated drugs at various concentrations. After 48 h incubation, cells were lysed by CellTiter Glo reagent (Promega, #G7572) and the luminescence signals were detected through a multimode microplate reader (VICTOR Nivo).

### Cell apoptosis assay

After treated with different concentrations of GNF-7, gilteritinib or AC220 for 48 h, leukemia cells were harvested and incubated with Annexin V-FITC and PI (KeyGEN BioTECH, China). The portion of Annexin V^+^ cells were detected by flow cytometry (BD Bioscience, San Jose, CA, USA).

### Western blotting

Western blotting was performed as previously described [15]. Briefly, cell samples were harvested and lysed by 1 × SDS lysis buffer. Equal amount of protein samples was loaded on polyacrylamide gel and then transferred to nitrocellulose membrane. The membrane was then blotted with specific primary antibodies against p-FLT3 (Tyr591, #3466, CST), p-AKT (Ser473, #4060S, CST), ERK (#4695S, CST), p-ERK (T202/Y204, #4370S, CST), p-Stat5 (Y694, #AP0887, Abclonal), FLT3 (#ab245116, Abcam), and Stat5 (#13179-1-AP, Proteintech), AKT (#10176-2-AP, Proteintech), actin-HRP (#HRP-60008, Proteintech). After overnight incubation at 4℃, HRP-conjugated secondary antibodies were applied and luminescence signals on membrane were detected with electrochemical luminescence (BIO-RAD).

### Cellular thermal shift assay (CETSA)

CETSA assay was performed as previously described [16]. Ba/F3 FLT3-ITD cells were treated with GNF-7 (1 μM) or DMSO for one hour, then harvested and lysed by liquid nitrogen. The lysates were divided into equal volume and heated at different temperatures for three minutes, after cooled to room temperature, lysates were centrifuged and the supernatants were collected and subjected to SDS-PAGE and western blotting analysis.

The dose effect of GNF-7 on the thermal stability of FLT3 was evaluated as follows. Same number of Ba/F3 FLT3-ITD cells were exposed to various concentrations of GNF-7 for one hour and then lysed by liquid nitrogen. Subsequently, the lysate solutions were heated at 50 °C for three min and then centrifuged, and the supernatants were subjected to SDS-PAGE and western blotting analysis.

### Animal models

0.8 × 10^6^ Ba/F3 FLT3-ITD cells or 0.5 × 10^6^ Ba/F3 FLT3-ITD-F691L cells were injected intravenously into BALB/c mice (6–8 weeks old, female, purchased from Beijing Vital River Laboratory Animal Technology Co., Ltd.), respectively. The mice were randomly divided into four groups. Three days after the cell injection, the treatment group received GNF-7, gilteritinib, AC220 or the same volume of solvent. Oral dosing with 15 mg/kg GNF-7 was administered to the mice once a day, 10 mg/kg AC220 and 30 mg/kg gilteritinib were used as the control. In order to assess the effect of the therapy, leukemia cells infiltrating peripheral blood or bone marrow was collected from each group of mice and analyzed by flow cytometry. Moreover, the leukemia burden of mice model was also measured by the spleen weight. The survival time of the mice was determined.

In the patient-derived xenograft (PDX) model, five weeks old female NOG mice (Charles River) were sub-lethally administrated with busulfan (30 mg/kg) before tail vein injection of AML #3 or AML #4 primary cells. After engraftment was successfully established, the primary blasts (2 × 10^6^ human AML #3 cells or 5 × 10^5^ human AML #4 cells) were collected, and then reinjected into busulfan treated NOG mice for the secondary transplantation. Soon afterwards, the transplanted mice were randomly divided into three groups. 15 days or 44 days after the engraftment, AML #3 or AML #4 cells transplanted mice was oral administrated with vehicle, 30 mg/kg gilteritinib and 15 mg/kg GNF-7, respectively. Residual leukemia cells were identified with hCD45 antibody (Biolegend) and leukemia stem and progenitor cells were identified with hCD45 plus with hCD34 antibody (BD Bioscience) through flow cytometry.

Animal experiments were conducted in accordance with established guidelines and were approved by the Institutional Animal Care and Welfare Committee of Guangzhou Women and Children’s Medical Center, Guangzhou Medical University.

### Model of GNF-7 bound to FLT3 protein

AutoDock Vina 1.1.2 software was used for molecular docking work, and the structure of the small molecule GNF-7 was energy minimized using AVOGADR 1.2.0 under the MMFF94 force field before the docking began. The FLT3 protein (5X02) was hydrotreated using PyMol software. Then ADFRsuite 1.0 was used to convert small molecules and receptor proteins into PDBQT format necessary for AutoDock Vina 1.1.2 docking. Before docking, center the box with the ATP site of the FLT3 protein was required. The detail of the global search for docking was set to 32, and the rest of the parameters remained the default settings. Finally, the highest-scoring docked conformation output was regarded as binding conformation and the docking results were visualized using PyMol and Ligplot 2.2.4 software.

### Statistical analysis

GraphPad Prism 8.0 software was used for statistical data analysis. Two-tailed paired Student’s *t* test was used for mean comparison between two groups, whereas the Kaplan–Meier survival curve and log-rank test were used for survival analysis. *P* values < 0.05 were considered statistically significant, and different levels were denoted as *, *P* < 0.05, **, *P* < 0.01, and ***, *P* < 0.001, respectively.

## Results

### GNF-7 selectively inhibits the proliferation of FLT3-ITD AML cells

**Fig. 1:**
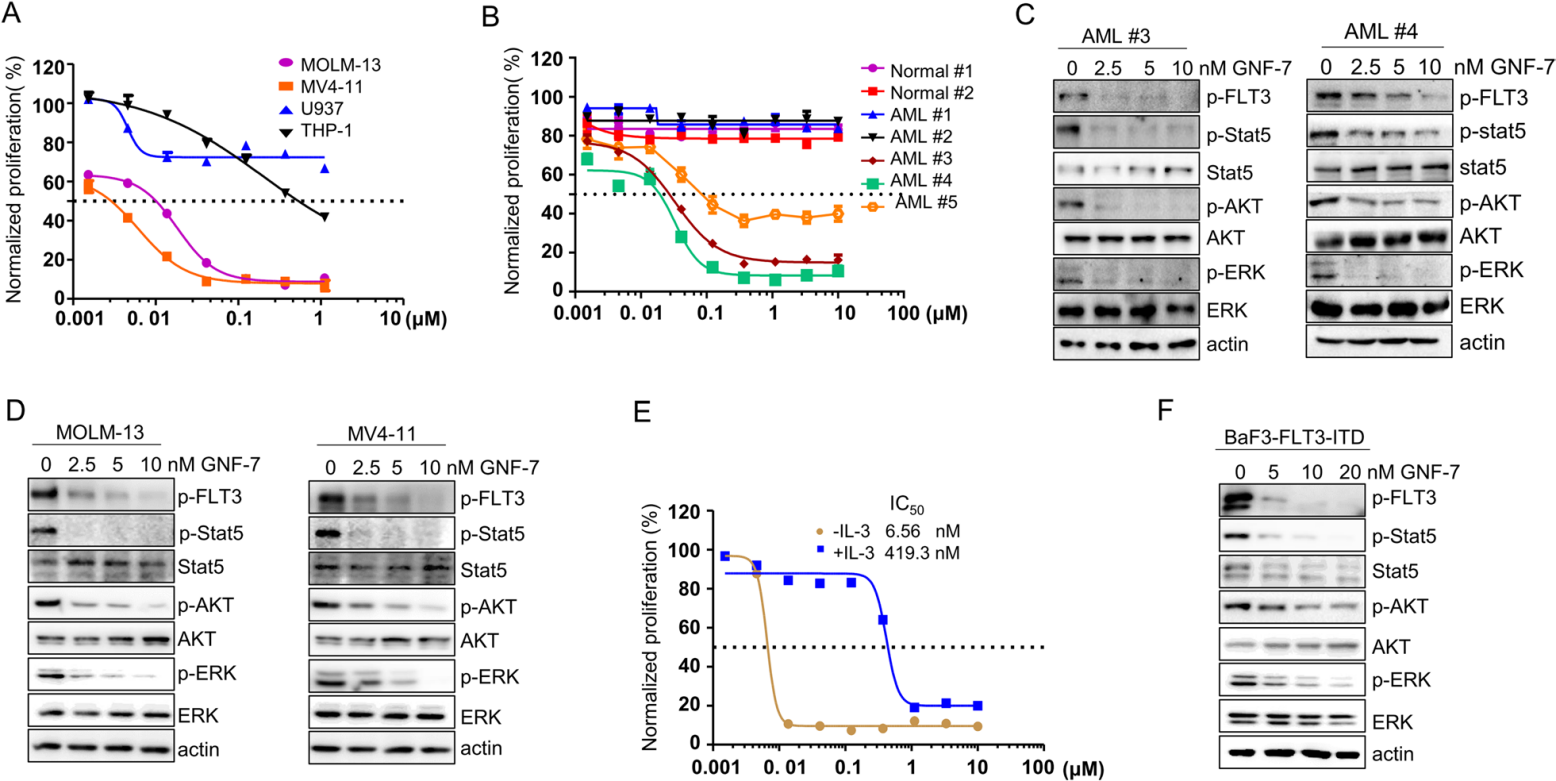
GNF-7 potently inhibited the proliferation of FLT3-ITD AML cells and targeted FLT3-ITD downstream signaling pathways. **A** AML cell line MOLM-13, MV4-11, U937 and THP-1 were treated with DMSO or increasing concentrations of GNF-7 for 48 h and the normalized cell proliferation was measured by CellTiter Glo assay. **B** Mononuclear cells isolated from umbilical cord blood (Normal #1, Normal #2), bone marrow of diagnosed with AML (AML #1, AML #2) and AML harboring FLT3-ITD mutation (AML #3, AML #4, AML #5) were treated with different concentrations of GNF-7 for 48 h, and normalized cell proliferation was detected by CellTiter Glo assay. **C** Primary bone marrow cells isolated from AML #3 and AML #4 were exposed to GNF-7 for 4 h and then detected by western blotting with antibodies against phosphorylated and total of FLT3, Stat5, AKT and ERK, respectively. **D** Phosphorylated and total of FLT3, Stat5, AKT and ERK in MOLM-13 and MV4-11 cells treated with GNF-7 for 4 h were detected by western blotting. **E** Dose response curve of GNF-7 on Ba/F3 FLT3-ITD cells in the presence or absence of IL-3. **F** Ba/F3 FLT3-ITD cells were treated with the different concentration of GNF-7 for 4 h and subjected to western blotting with the indicated antibodies. All experiments were repeated three times with the same results. Data are presented as mean ± SD, and *P* values were calculated using Student t test. *p < 0.05, ** *p* < 0.01, and *** *p* < 0.001

As previously reported, GNF-7 functions as a BCR::ABL1 inhibitor that can overcome the “gatekeeper” BCR::ABL1/T315I mutation that resistant to tyrosine kinase inhibitors (TKIs) [13]. Indeed, GNF-7 potently inhibited the proliferation of BCR::ABL1/T315I expressing BaF3 cells that resistant to the BCR::ABL1 inhibitors imatinib and dasatinib (Additional file 1: Fig. S1A–C) [17]. Interestingly, we found that GNF-7 showed potent anti-proliferation on Ba/F3 cells stably express FLT3-ITD, whereas TKIs such as imatinib and dasatinib exhibited no significant proliferation inhibition (Additional file 1: Fig. S2). To assess the anti-proliferative activity of GNF-7 in AML cell lines, we focused on the human AML cell lines harboring FLT3-ITD mutations or FLT3-WT. Interestingly, GNF-7 preferentially inhibited cell proliferation of FLT3-ITD-dependent leukemia cells (MOLM-13 and MV4-11) in a dose-dependent manner, whereas it has no apparent cytotoxic effect on leukemia cells with FLT3-WT (THP-1 and U937) in low dose concentration (Fig. 1A). We then collected primary mononuclear cells from two umbilical cord blood samples (Normal #1, Normal #2), primary bone marrow mononuclear cells from two AML patients diagnosed with none FLT3-ITD mutation AML (AML #1, AML #2) and three AML patients harboring FLT3-ITD mutation (AML #3, AML #4, AML #5) to extensively evaluate the anti-FLT3-ITD AML activity of GNF-7. GNF-7 exerted very potent inhibitory effect on the proliferation of FLT3-ITD AML leukemia cells (AML #3, AML #4, AML #5) compared with normal mononuclear cells or none FLT3-ITD mutation AML cells (Fig. 1B). Particularly, GNF-7 showed more anti-proliferative effect on Ba/F3 FLT3-ITD cells compared with Ba/F3 FLT3-ITD cells treated with IL-3 (6.56 nM vs 419.3 nM) (Fig. 1E). FLT3-ITD or FLT3-TKD mutants undergo constitutive autophosphorylation of FLT3, causing aberrant activation of downstream pathways such as STAT5, PI3K/AKT and MAPK/ERK [4, 5]. Consistent with the significant proliferation inhibition of FLT3-ITD harboring cells, the phosphorylation of downstream effectors in the FLT3 signaling pathways, such as p-Stat5, p-AKT and p-ERK1/2 was also significantly inhibited by GNF-7 in AML patients harboring FLT3-ITD mutation, FLT3-ITD-dependent AML cells, and Ba/F3 FLT3-ITD cells, respectively (Fig. 1C, D, F).

### GNF-7 interact with FLT3 protein

**Fig. 2:**
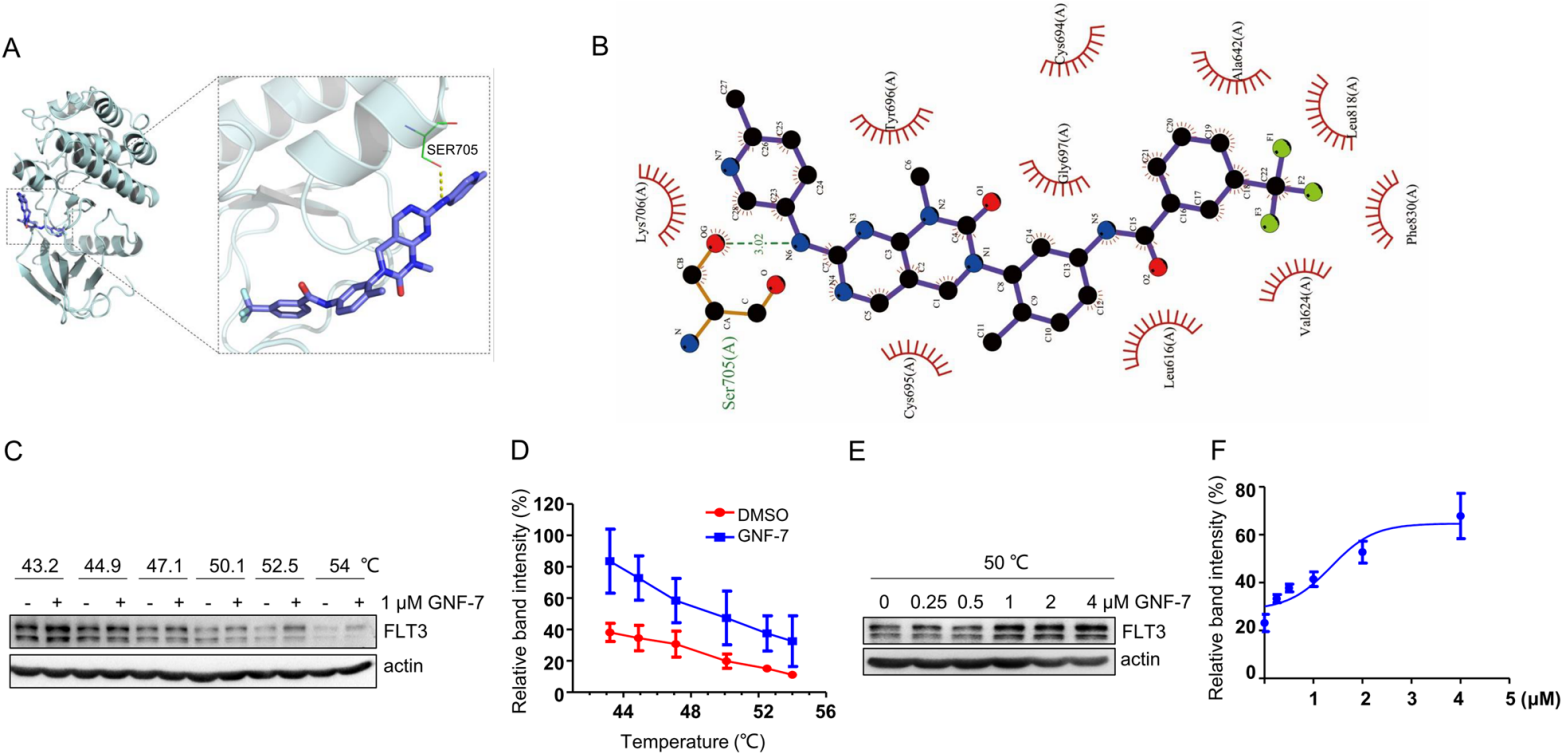
GNF-7 interacts with FLT3 protein. **A** Docking model of FLT3 bound to GNF-7: the yellow dotted line represents the hydrogen bond interaction, the green line represents the amino acid that forms hydrogen bonds with GNF-7, the cartoon represents the FLT3 protein, and the purple stick represents the GNF-7 molecule. **B** 2D interaction diagram of the FLT3/GNF-7 complex: GNF-7 is bound to the FLT3 protein in a pocket surrounded by LYS706, TYR696, GLY697, CYS694, ALA642, LEU818, PHE830, VAL624, LEU616, CYS695, and SER705 amino acids, GNF-7 forms hydrogen bonds with SER705 and forms hydrophobic interaction with LYS706, TYR696, GLY697, CYS694, ALA642, LEU818, PHE830, VAL624, LEU616, CYS695. **C**, **E** Thermal stabilization of FLT3 in Ba/F3 FLT3-ITD cells treated with GNF-7 (1 μM) or DMSO in various temperatures (**C**) and treated with various concentrations of GNF-7 (**E**) was analyzed through CETSA assay. **D**–**F** The density of the FLT3 bands were quantified by quantity one software. All experiments were repeated three times with the same results. Data are presented as mean ± SD, and *P* values were calculated using Student t test. * *p* < 0.05, ** *p* < 0.01, and *** *p* < 0.001

To understand the potential structural effects of FLT3 on GNF-7 binding, we used a docking model of GNF-7 bound to FLT3, based on the crystal structure, we found that GNF-7 forms hydrogen bonds with SER705 of FLT3 protein (Fig. 2A). In addition, a 2D interaction diagram of the FLT3/GNF-7 complex clearly showed that GNF-7 forms an interaction network with LYS706, TYR696, GLY697, CYS694, ALA642, LEU818, PHE830, VAL624, LEU616, CYS695 via hydrophobic interaction (Fig. 2B). The cellular thermal shift assay (CETSA) was a novelly developed and wildly used method for detecting drug-binding to target proteins in cells or tissue samples [14, 18]. CETSA was further used to confirm the interactions between GNF-7and FLT3. Compared with DMSO, addition of GNF-7 increased the thermal stability of FLT3 in different temperature exposure (Fig. 2C, D). Moreover, the thermal stability of FLT3 protein was increased by GNF-7 in a dose-dependent manner (Fig. 2E, F). These results demonstrated that GNF-7 directly interacts with FLT3.

### GNF-7 exhibits effective anti-FLT3-ITD positive AML cells activity in mouse model

**Fig. 3:**
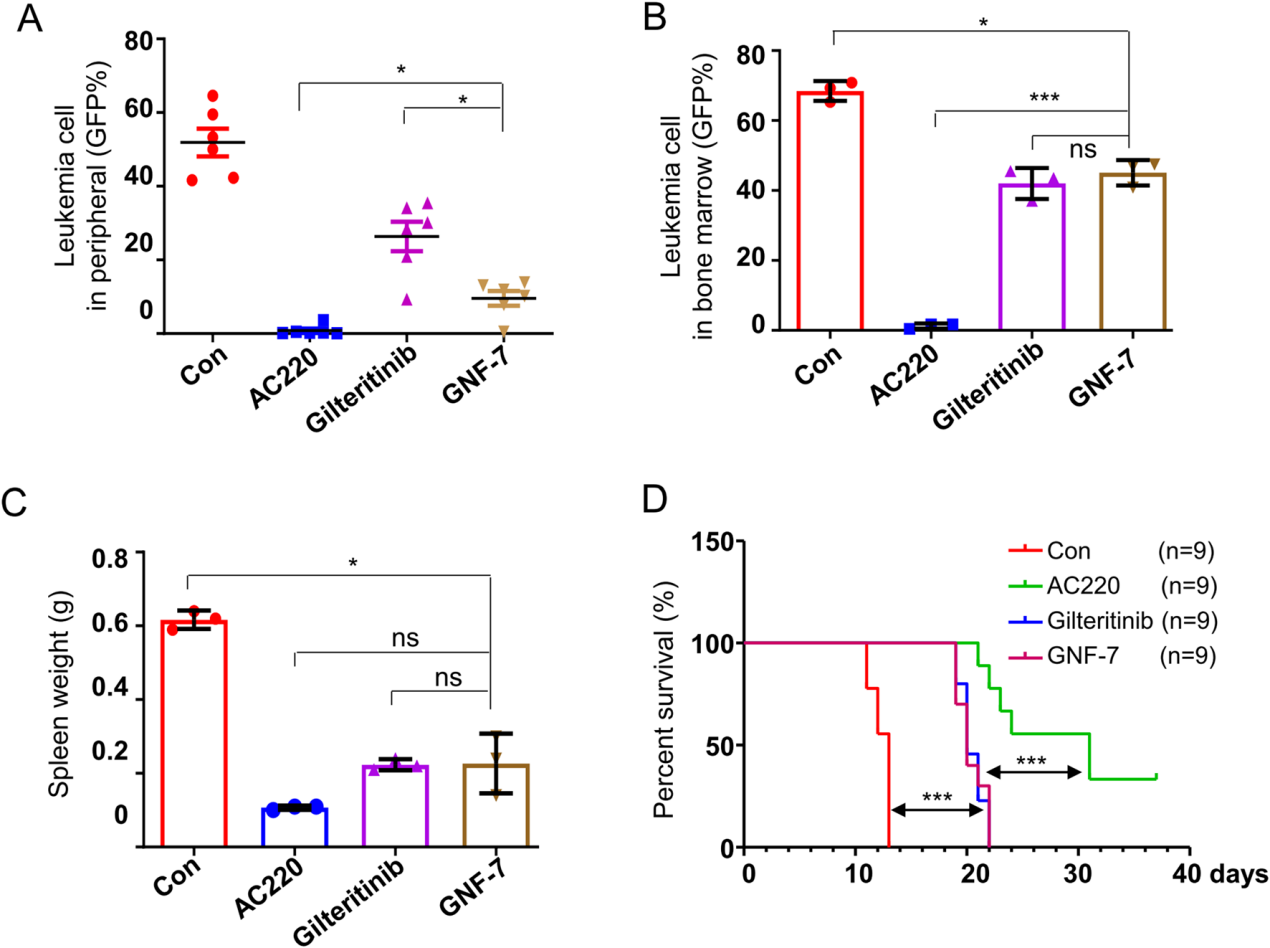
GNF-7 showed significant therapy effect on the mice model engrafted with Ba/F3 FLT3-ITD cells. **A** The mice engrafted with Ba/F3 FLT3-ITD cells were treated with vehicle, AC220 (10 mg/kg), gilteritinib (30 mg/kg) and GNF-7 (15 mg/kg) for 8 days and the percentages of leukemia cells infiltrated in peripheral blood were then analyzed by flow cytometry. **B** The mice engrafted with Ba/F3 FLT3-ITD cells were treated with vehicle, AC220 (10 mg/kg), gilteritinib (30 mg/kg) and GNF-7 (15 mg/kg) for 9 days and the percentages of leukemia cells infiltrated in bone marrow were then analyzed by flow cytometry. **C** The spleen weight in each group were measured. **D** The survival curve of mice was calculated. Data are presented as mean ± SD, and *P* values were calculated using Student t test. * *p* < 0.05, ** *p* < 0.01, and *** *p* < 0.001

In order to evaluate GNF-7 efficacy in vivo, we generated a mice model of FLT3-ITD positive AML through intravenously inject Ba/F3 FLT3-ITD cells. To assess the anti-tumor efficacy of GNF-7, FDA approved drug for relapsed/refractory AML [19]—gilteritinib and a highly potent type II FLT3 inhibitor—AC220 [8] were used as a measure of positive drug efficacy. After therapy for 8 days, GNF-7 significantly reduced the leukemia cells in peripheral blood (51.9% in the vehicle administration group, 26.4% in the gilteritinib administration group vs 9.6% in the GNF-7 administration group) compared with vehicle administration group (Fig. 3A). ACC20 exhibits the most potent effect on reducing the infiltration of leukemia cells into peripheral blood (0.8% in the AC220 administration group). Three mice were randomly selected from each group and the burden of leukemia cells in bone marrow or spleen were analyzed after 9 days treatment. Compared with vehicle administration group, GNF-7 potently reduced the proportion of leukemia cells in bone marrow (68.5% in the vehicle administration group vs 45.0% in the GNF-7 administration group) and showed no obvious difference on reducing the leukemia cells as gilteritinib (Fig. 3B). ACC20 showed the most potent effect on reducing the infiltration of leukemia cells into bone marrow (1.3% in the AC220 administration group). Furthermore, Gilteritinib significantly reduced the spleen weight compared with vehicle group, and no significant difference was observed in spleen weight changes among AC220, gilteritinib and GNF-7 groups (Fig. 3C). It suggests that GNF-7 has no less potency than gilteritinib against leukemia cells infiltration. We next measured the survival of each group mice and the result showed that GNF-7 exhibited similar effect with gilteritinib on significantly prolonged the survival of mice (Fig. 3D). Above result demonstrated that although the anti-FLT3-ITD leukemia cells effect in vivo was weaker than AC220, GNF-7 showed the equal effect as gilteritinib.

### GNF-7 overcomes FLT3-ITD/F691L drug resistance

**Fig. 4:**
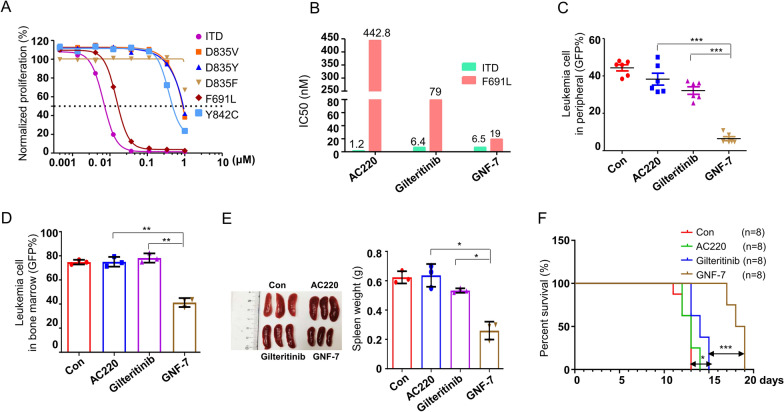
Activity of GNF-7 against drug resistant Ba/F3 FLT3-ITD/F691L cells. **A** Relative proliferation of Ba/F3 populations stably expressing FLT3-ITD mutant isoforms after 48 h in various concentrations of GNF-7 were measured by CellTiter Glo assay. **B** IC_50_ values of Ba/F3 stably expressing FLT3-ITD and FLT3-ITD/F691L cells treated with various concentrations of AC220, gilteritinib and GNF-7 were analyzed by CellTiter Glo assay. **C** After treated with vehicle, AC220 (10 mg/kg), gilteritinib (30 mg/kg) and GNF-7 (15 mg/kg) for 8 days, the percentages of leukemia cells infiltrated in peripheral blood of mice (n = 6) engrafted with Ba/F3 FLT3-ITD/F691L cells were evaluated by flow cytometry. **D** After treated with vehicle, AC220 (10 mg/kg), gilteritinib (30 mg/kg) and GNF-7 (15 mg/kg) for 9 days, the burden of leukemia cells in bone marrow of mice (n = 3) which was randomly selected from each group were detected. **E** The spleen weights were analyzed. **F** The Kaplan–Meier survival curves of animal survival of mice treated with vehicle, AC220 (10 mg/kg), gilteritinib (30 mg/kg) and GNF-7 (15 mg/kg). All cell line experiments were repeated three times with the same results. *P* values were calculated by log-rank test and shown. Data are presented as mean ± SD, and *P* values were calculated using Student t test. * *p* < 0.05, ** *p* < 0.01, and *** *p* < 0.001

FLT3 mutants identified de novo in AML patients causing drug resistance. We then sought to determine whether GNF-7 showed inhibitory activity in AC220 resistant Ba/F3 cells that stably expressing FLT3-ITD/D835V, FLT3-ITD/D835Y, FLT3-ITD/D835F, FLT3-ITD/Y842C and FLT3-ITD/F691L [8, 20]. GNF-7 potently inhibited the growth of Ba/F3 FLT3-ITD cells and retained strong inhibitory activity against the Ba/F3 FLT3-ITD/F691L cells (Fig. 4A), which was the main drug resistance mode in the therapy of FLT3 inhibitors. Notably, the proliferation inhibitory potency of GNF-7 on Ba/F3 FLT3-ITD/F691L cells was 23 folds than that of AC220 (19 nM vs 442.8 nM), and was 4 folds than that of gilteritinib (19 nM vs 79 nM) (Fig. 4B). In addition, GNF-7 induced a remarkable level of apoptosis in Ba/F3 FLT3-ITD/F691L cells in a dose dependent manner, whereas, AC220 and gilteritinib had no significant effect on cell apoptosis at the same concentrations (Additional file 1: Fig. S3).

Next, we established the previously described Ba/F3 FLT3-ITD/F691L leukemia model^11^ to evaluate the efficacy of GNF-7 in vivo. After treated with GNF-7 for 8 days, leukemia cells infiltration in peripheral blood were significantly reduced compared with other three groups (Fig. 4C). Interestingly, with nearly 50% leukemia cells infiltrated in bone marrow were eliminated after 9 days of GNF-7 treatment, but AC220 and gilteritinib did not show significant effect (Fig. 4D). The average spleen mass of mice in group vehicle, AC220, gilteritinib and GNF-7 was 0.62 g, 0.64 g, 0.53 g and 0.26 g respectively, suggesting that GNF-7 also largely reduced the infiltration of leukemia cells in the spleen (Fig. 4E). Consistent with the results of leukemia cells infiltrating in various organs, GNF-7 significantly prolonged the survival period of mice compared with gilteritinib group or AC220 group (Fig. 4F), whereas, AC220 and gilteritinib slightly prolonged the survival period of mice. These data suggested that GNF-7 can overcome FLT3-ITD/F691L drug resistance for the treatment of AML in *vivo* and in vitro.

### GNF-7 significantly prolonged the survival of FLT3-ITD AML PDX model mice

**Fig. 5:**
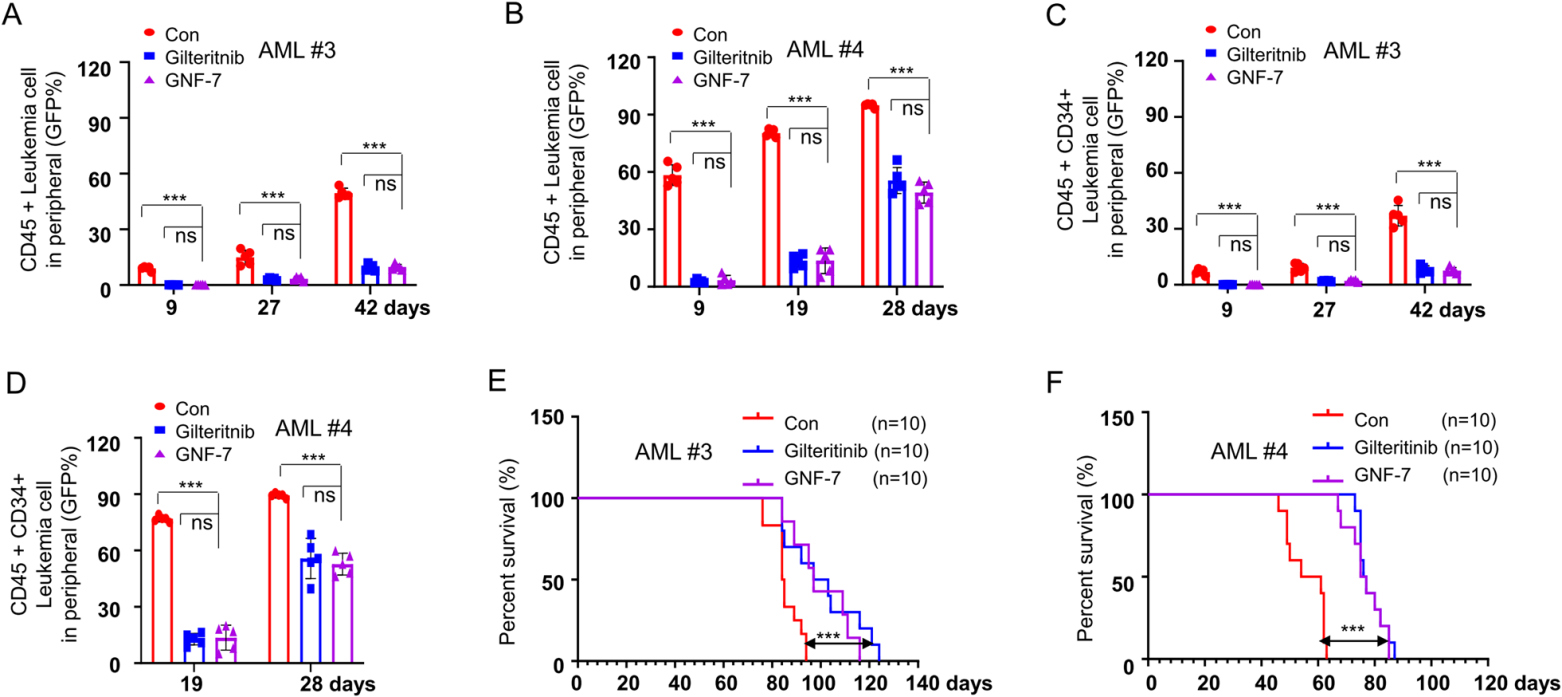
GNF-7 exerts potent therapy effect on AML PDX model. **A**, **B** Primary patient cells from AML #3 or AML #4 carrying with FLT3-ITD were transplanted into busulfan pretreated NOG mice and then randomly divided into three groups. Peripheral blood of 5 mice administrated with vehicle, 15 mg/kg GNF-7 and 30 mg/kg Gilteritinib were collected at the indicated time, and the leukemia cells content was detected by flow cytometry using human CD45 antibody. **C**, **D** The percentages of leukemia stem and progenitor cells in peripheral blood were detected by flow cytometry using human CD45 and CD34 antibodies. **E**, **F** The Kaplan–Meier survival curves of animal survival of mice treated with vehicle, AC220 (10 mg/kg), gilteritinib (30 mg/kg) and GNF-7 (15 mg/kg)

Furthermore, we also collected AML primary cells from one FLT3-ITD AML patient (AML #5) and two FLT3-ITD AML relapsed patients (Additional file 1: Table S1) to test the anti-leukemia effects of GNF-7. Gilteritinib was a potent FLT3 inhibitor drug with single-agent activity in relapsed or refractory FLT3-ITD AML and achieved satisfactory prognosis [19]. Compared with gilteritinib, the same concentration of GNF-7 had a stronger inhibitory effect on leukemia cell proliferation in three FLT3-ITD AML patients (Additional file 1: Fig. S4A–C). In order to further clarify the therapeutic effect of GNF-7 in vivo, primary bone marrow leukemia cells from of AML#3 and AML#4 were selected to establish PDX models to evaluate the efficacy of GNF-7. Both GNF-7 and gilteritinib significantly reduced the infiltration of AML blast cells that expressed with CD45 [21] in the peripheral blood (Fig. 5A, B), with no significant difference in effect. Leukemic stem cells are considered the origin of relapse and insensitive to the cytotoxicity of the conventional chemotherapy in AML [22]. CD45 is expressed in all nucleated cells, and CD34 is expressed in hematopoietic stem and progenitor cells. Interestingly, we found that GNF-7 and gilteritinib also showed potent cytotoxic effect on leukemic stem and progenitor cells (Fig. 5C, D). In addition, GNF-7 had the same effect as gilteritinib on reducing leukemia cells invasion in the spleen, such as both significantly reduced spleen weight and leukemia cell burden (Additional file 1: Fig. S5A–D). Notably, GNF-7 significantly prolonged the survival of mice and showed no significant difference compared with gilteritinib (Fig. 5E, F). These results suggest that GNF-7 does indeed exhibit favorable therapy in FLT3-ITD AML PDX model and its effects is similar to those of gilteritinib.

Taken together, we show that GNF-7 is a very potent FLT3 inhibitor that exerts strong anti-leukemia effects against AML cells harboring FLT3-ITD and FLT3-ITD/F691L both in vitro and in vivo, which is recognized as the difficult mutation to overcome clinically. Additionally, our results also show that GNF-7 may be an effective therapeutic compound in FLT3-ITD AML relapsed patients. Considering the effective treatment, GNF-7 may become a promising second-line drug suitable for treating some of the most clinically challenging AML cases.

## Discussion

AML is a heterogeneous disease, characterized by a wide range of genomic changes and molecular mutations that affect clinical prognosis. FLT3-ITD/TKD is a frequent gene mutation occurs in AML, which accounts for a large proportion of AML patients [1]. FLT3-ITD not only can be used as a prognosis indicator in AML, but also recognized as a molecular marker of minimal residual disease in detecting the progress of FLT3-ITD-AML [23]. Targeted therapy for FLT3-ITD significantly improved the survival of AML patients, thus, FLT3 inhibitors are popularly developed. Unfortunately, drug-resistant mutations in the therapy of FLT3 inhibitors frequently occurred and make the new FLT3 inhibitors more required.

GNF-7 is a BCR::ABL1 inhibitor that can override T315I “gatekeeper” mutation and other BCR::ABL1 mutants [13]. In addition, we found that GNF-7 could also selectively inhibited the proliferation of AML cells that expressing FLT3-ITD in vitro and in vivo, and potently inhibited FLT3-ITD downstream signaling pathways. Structure model and CETSA experiment demonstrated that GNF-7 could bind with FLT3, further indicated that GNF-7 is a potent FLT3 inhibitor. Kinase profiling revealed that GNF-7 not only evidently inhibited LYN, FYN, SRC, YES, BTK and CSK kinase that can also inhibited by dasatinib [24], but also inhibited ACK1 and mitogen-activated protein kinase (GCK) to kill NRAS-dependent cells in AML and acute lymphoblastic leukemia [25]. Moreover, GNF-7 induced diffuse large B-cell lymphoma (DLBCL) cell apoptosis through suppressing GCK activation [26]. These above researches suggest that the characteristics of multitargeted kinase confers GNF-7 more promising in the treatment of leukemia.

The acquired secondary FLT3-ITD mutants especially at F691L was acquired in some patients [27] and was identified to confer resistance to most FLT3 inhibitors, such as AC220, sorafenib and gilteritinib [10, 28]. Ponatinib was a potent tyrosine kinase inhibitor of BCR::ABL1 and mutated BCR::ABL1, including BCR::ABL1/T315I and was also reported to have mild inhibitory activity against FLT3-ITD/F691L [29, 30]. In this study, we demonstrated potent inhibitory activity of GNF-7 against FLT3-ITD/F691L in vitro*.* Compared with AC220 and gilteritinib, GNF-7 showed more potent therapeutic effect on the treatment of FLT3-ITD/F691L expressing cells in vivo. Considering that AML cell lines may have limitations in reflecting the effect of FLT3 inhibitors, we established two FLT3-ITD AML relapsed patient-derived xenograft model to further evaluate the anti-FLT3-ITD AML potency of GNF-7. Compared with gilteritinib, GNF-7 exerted similar effect of killing leukemic stem cells and prolonging the survival of PDX model.

## Conclusion

Our investigation has revealed that GNF-7 is a novel FLT3 inhibitor which shows potent anti-leukemic activity in FLT3-ITD AML. Furthermore, we have demonstrated that GNF-7 overcomes FLT3-ITD/F691L drug resistance. Based on these finding, our study provides supporting evidence and a basis for GNF-7 could be the promising drug candidate for the treatment of AML cells with various FLT3 mutations.

### Supplementary Information

**Additional file 1: Table S1. Figure S1.** GNF-7 overcomes BCR-ABL/T315I resistance. Normalized cell proliferation of Ba/F3 P190 and Ba/F3 T315I cells treated with various concentrations of imatinib (A), dasatinib (B) and GNF-7 (C) for 48 hours was measured by the CellTiter Glo assay. Data are presented as mean ± SD, and *P* values were calculated using Student t test. * *p *< 0.05, ** *p* < 0.01, and *** *p* < 0.001. **Figure S2.** GNF-7 significantly inhibited the proliferation of Ba/F3 FLT3-ITD cells. Ba/F3 FLT3-ITD cells were treated with various concentrations of imatinib, dasatinib and GNF-7 for 48 hours. CellTiter Glo assay was applied to measure the normalized cell proliferation of these cells. Data are presented as mean ± SD, and *P* values were calculated using Student t test. * *p *< 0.05, ** *p* < 0.01, and *** *p* < 0.001. **Figure S3.** GNF-7 significantly induced Ba/F3 FLT3-ITD/F691L cells apoptosis. After treated with the same concentration of AC220, gilteritinib and GNF-7 for 48 hours, the apoptosis rate of Ba/F3 FLT3-ITD/F691L cells were analyzed by flow cytometry. Data are presented as mean ± SD, and *P* values were calculated using Student t test. * *p *< 0.05, ** *p* < 0.01, and *** *p* < 0.001. **Figure S4.** GNF-7 have potent therapy effect on FLT3-ITD harboring AML. (A-C) Primary bone marrow cells isolated from 3 diagnosed FLT3-ITD AML patients were treated with gilteritinib and GNF-7 for 48 hours and the normalized cell proliferation was measured by the CellTiter Glo assay. Data are presented as mean ± SD, and *P* values were calculated using Student t test. * *p *< 0.05, ** *p* < 0.01, and *** *p* < 0.001. **Figure S5.** GNF-7 inhibits the infiltration of primary blasts in PDX model. After treated with vehicle, GNF-7 and gilteritinib, three NOG mice transplanted with primary cells from AML #3 patient were randomly selected from each group and then analyzed spleen weight (A) and the content of leukemia cells in spleen were detected by flow cytometry using human CD45 antibody (B). C Spleen weight of NOG mice transplanted with primary cells from AML #4 patient in each group were calculated and the content of leukemia cells in spleen were detected by flow cytometry using human CD45 antibody, three mice were selected for each group (D). Data are presented as mean ± SD, and *P* values were calculated using Student t test. * *p *< 0.05, ** *p* < 0.01, and *** *p* < 0.001.

## Data Availability

The data that support the findings of this study are available on reasonable request from the corresponding author.

## Abbreviations

FLT-ITD: FMS-like tyrosine kinase 3 internal tandem duplication
FBS: Fetal bovine serum
AC220: Quizartinib
SDS: Sodium dodecyl sulfate
TKI: Tyrosine kinase inhibitor
DMSO: Dimethyl sulfoxide
FDA: Food and Drug Administration
PDX: Patient-derived xenograft.
CETSA: Cellular thermal shift assay
