# Supplementary material for: GNF-7, a novel FLT3 inhibitor, overcomes drug resistance for the treatment of FLT3‑ITD acute myeloid leukemia
Source: Cancer Cell Int. 2023 Nov 30;23:302. doi: 10.1186/s12935-023-03142-y (PMC10691066; doi:10.1186/s12935-023-03142-y)

**Additional Information for Manuscript “GNF-7, a novel FLT3 inhibitor, overcomes drug resistance for the treatment of FLT3‑ITD acute myeloid leukemia”**

Xinhua Xiao^1#*^, Peihong Wang^3#^, Weina Zhang^1^, Jiayi Wang^1^, Mansi Cai^1^, Yingli Wu^4*^, Hua Jiang^1*^ and Huizhuang Shan^2*^

^1^ Department of Hematology and Oncology, Guangzhou Women and Children’s Medical Center, Guangzhou Medical University, Guangzhou, 510623, China.

^2^ Laboratory Medicine, Guangdong Provincial People’s Hospital, Guangdong Academy of Medical Sciences, Guangzhou, Guangdong 510000, China.

^3^ Department of Hematology, Guangzhou First People's Hospital, South China University of Technology, Guangzhou, Guangdong 510000, China.

^4^ Hongqiao International Institute of Medicine, Shanghai Tong ren Hospital/Faculty of Basic Medicine, Chemical Biology Division of Shanghai Universities E-Institutes, Key Laboratory of Cell Differentiation and Apoptosis of the Chinese Ministry of Education, Shanghai Jiao Tong University School of Medicine, Research Units of Stress and Tumor(2019RU043), Chinese Academy of Medical Sciences, Shanghai, 200025 China.

^#^ These authors contributed equally to the work.

^*^ Corresponding Authors: Huizhuang Shan (shanhuizhuang@gdph.org.cn), Hua Jiang (jiang_hua18@sina.cn), Yingli Wu (wuyingli@shsmu.edu.cn), Xinhua Xiao (xinhxiao@163.com)

**This file includes:**

Table S1

Figures S1-S5

**Table S1**

| ID sample | Age | Gender | Source | Status | Genetic abnormalities |
| --- | --- | --- | --- | --- | --- |
| AML #1 | 12 | male | BM | dignostic | NRAS |
| AML #2 | 10 | female | BM | dignostic | NPM1 |
| AML #3 | 44 | male | BM | Relapsed | FLT3-ITD, CEBPA, DNMT3A |
| AML #4 | 60 | male | BM | Relapsed | FLT3-ITD, AXSL1, NF1 |
| AML #5 | 10 | female | BM | dignostic | FLT3-ITD, CBFB/MYH11gene rearrangement |

**Figure S1 GNF-7 overcomes BCR-ABL/T315I resistance.** Normalized cell proliferation of Ba/F3 P190 and Ba/F3 T315I cells treated with various concentrations of imatinib **(A)**, dasatinib **(B)** and GNF-7 **(C)** for 48 hours was measured by the CellTiter Glo assay. Data are presented as mean ± SD, and *P* values were calculated using Student t test. * *p* < 0.05, ** *p* < 0.01, and *** *p* < 0.001.

**
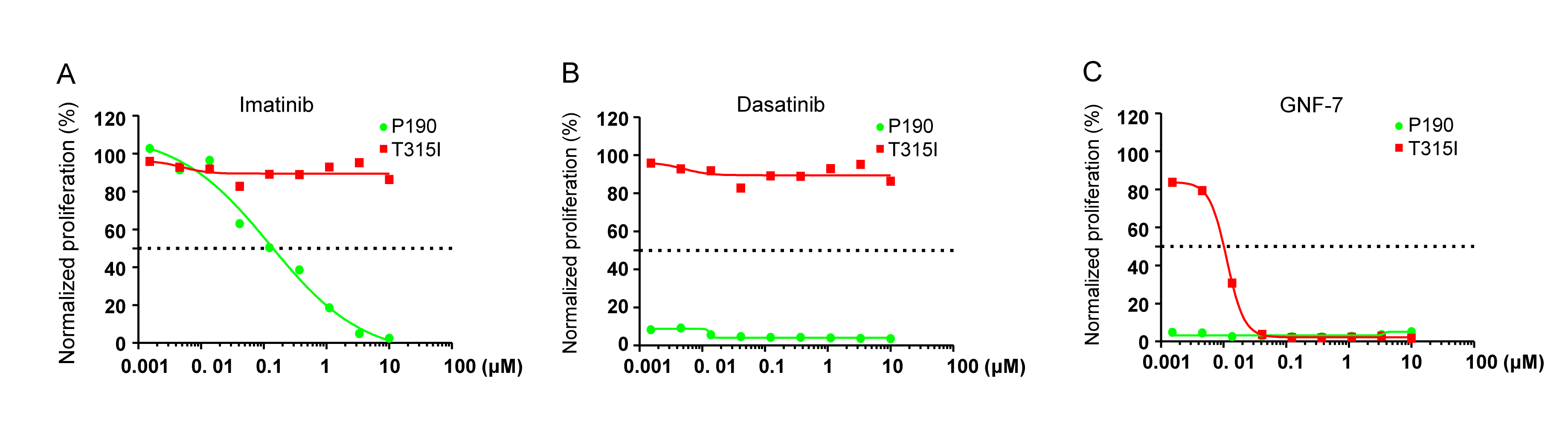
**

**Figure S2 GNF-7 significantly inhibited the proliferation of Ba/F3 FLT3-ITD cells.** Ba/F3 FLT3-ITD cells were treated with various concentrations of imatinib, dasatinib and GNF-7 for 48 hours. CellTiter Glo assay was applied to measure the normalized cell proliferation of these cells. Data are presented as mean ± SD, and *P* values were calculated using Student t test. * *p* < 0.05, ** *p* < 0.01, and *** *p* < 0.001.


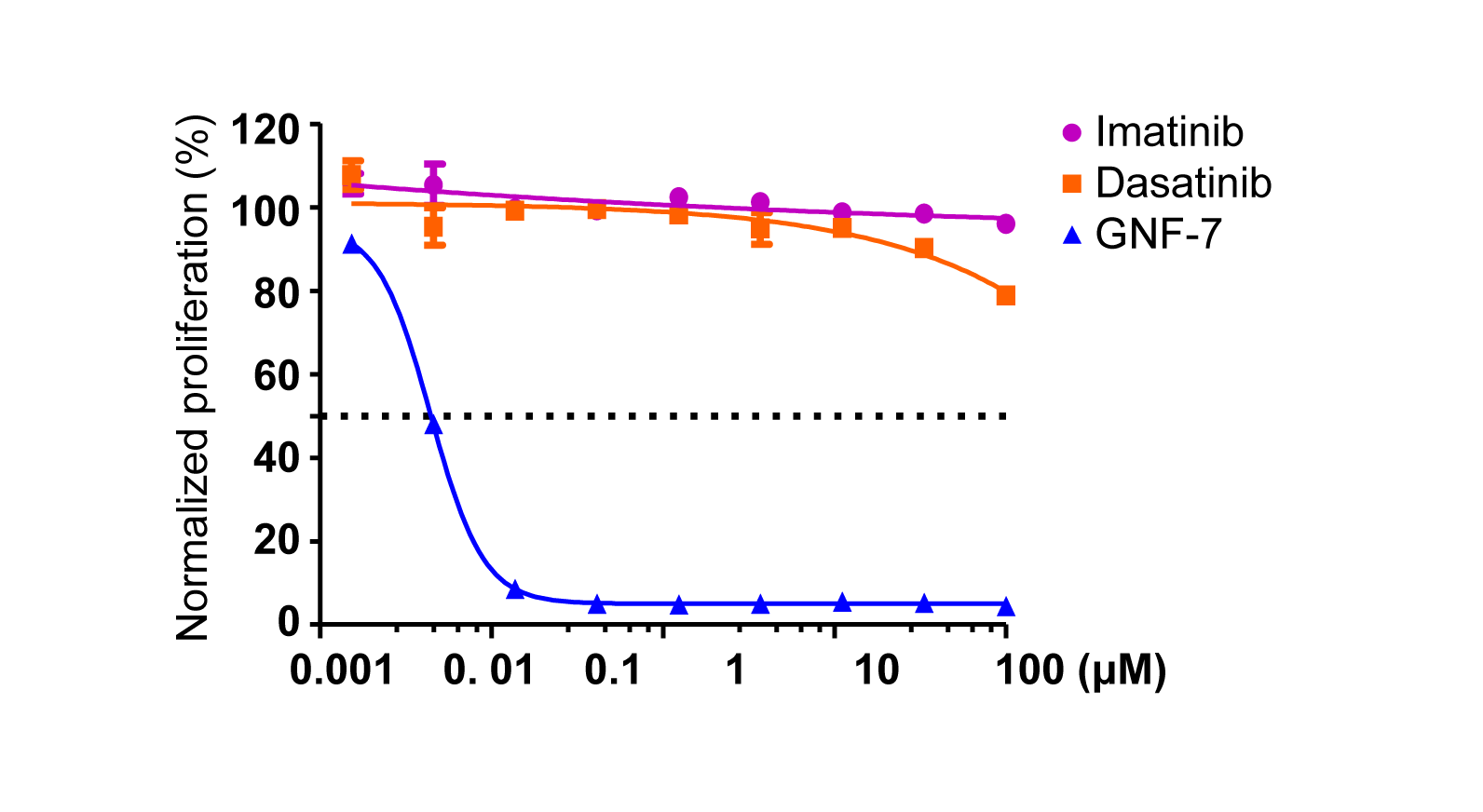


**Figure S3 GNF-7 significantly induced Ba/F3 FLT3-ITD/F691L cells apoptosis.** After treated with the same concentration of AC220, gilteritinib and GNF-7 for 48 hours, the apoptosis rate of Ba/F3 FLT3-ITD/F691L cells were analyzed by flow cytometry. Data are presented as mean ± SD, and *P* values were calculated using Student t test. * *p* < 0.05, ** *p* < 0.01, and *** *p* < 0.001.


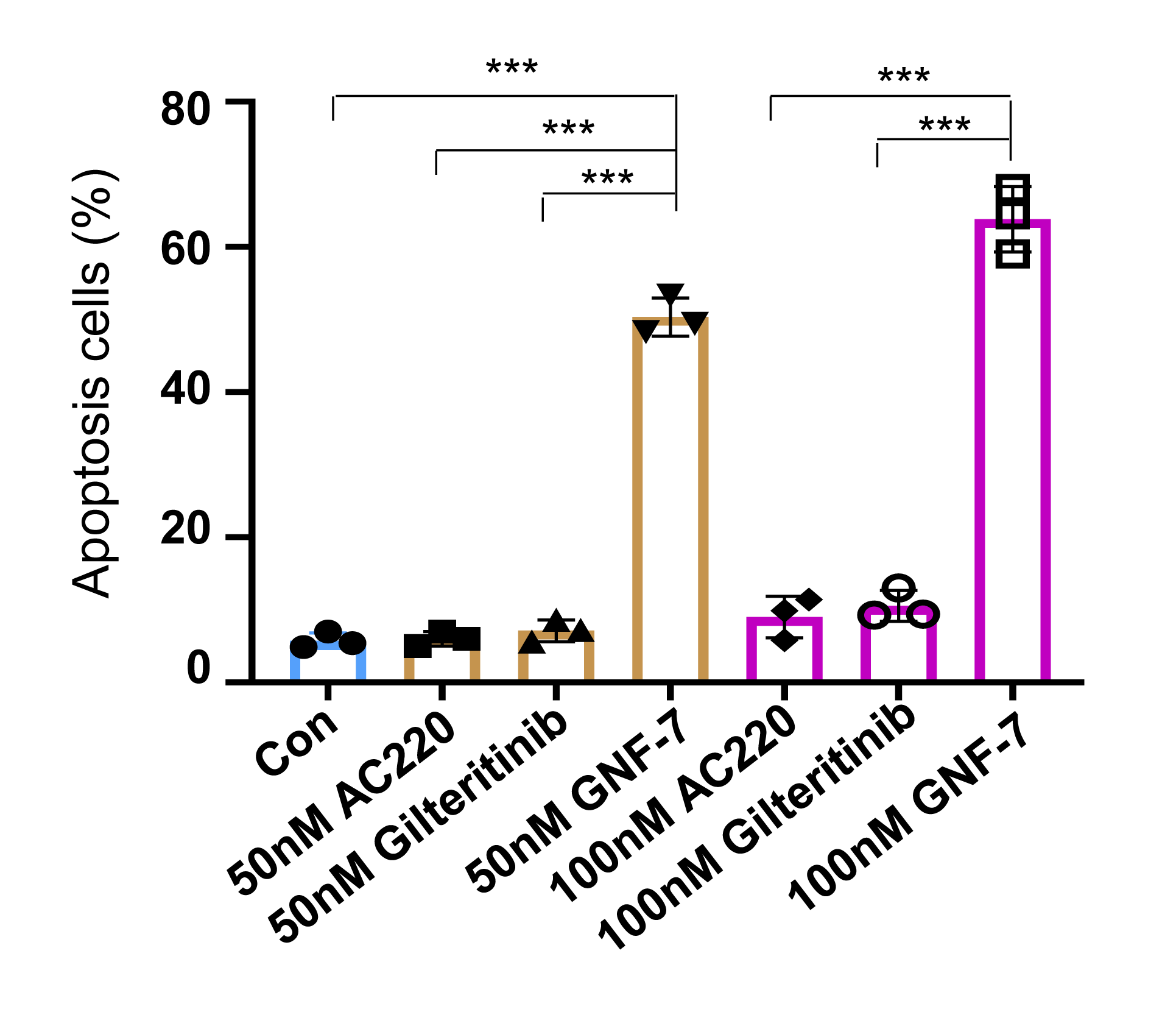


**Figure S4 GNF-7 have potent therapy effect on FLT3-ITD harboring AML.** **(A-C)** Primary bone marrow cells isolated from 3 diagnosed FLT3-ITD AML patients were treated with gilteritinib and GNF-7 for 48 hours and the normalized cell proliferation was measured by the CellTiter Glo assay. Data are presented as mean ± SD, and *P* values were calculated using Student t test. * *p* < 0.05, ** *p* < 0.01, and *** *p* < 0.001.


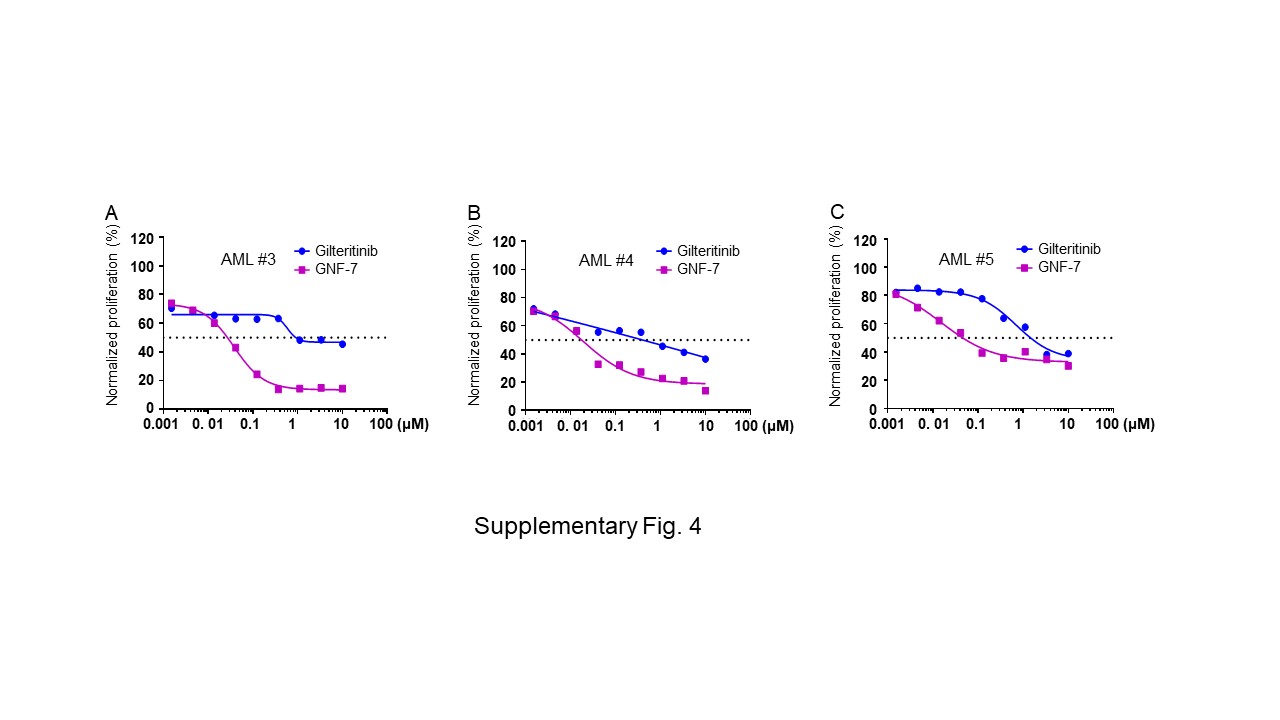


**Figure S5 GNF-7 inhibits the infiltration of primary blasts in PDX model.** After treated with vehicle, GNF-7 and gilteritinib, three NOG mice transplanted with primary cells from AML #3 patient were randomly selected from each group and then analyzed spleen weight **(A)** and the content of leukemia cells in spleen were detected by flow cytometry using human CD45 antibody **(B)**. **C** Spleen weight of NOG mice transplanted with primary cells from AML #4 patient in each group were calculated and the content of leukemia cells in spleen were detected by flow cytometry using human CD45 antibody, three mice were selected for each group **(D)**. Data are presented as mean ± SD, and *P* values were calculated using Student t test. * *p* < 0.05, ** *p* < 0.01, and *** *p* < 0.001.


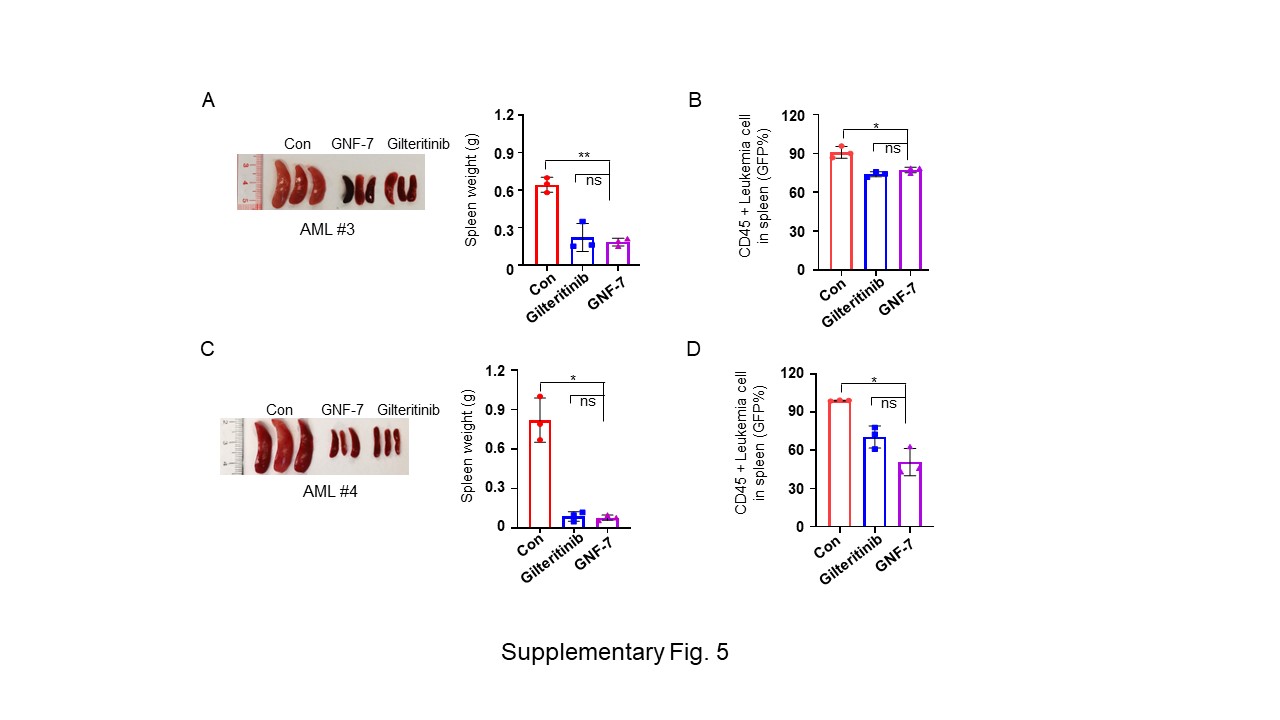

Supplement: Supplementary file 1 — Additional file 1: Table S1. Figure S1. GNF-7 overcomes BCR-ABL/T315I resistance. Normalized cell proliferation of Ba/F3 P190 and Ba/F3 T315I cells treated with various concentrations of imatinib (A), dasatinib (B) and GNF-7 (C) for 48 hours was measured by the CellTiter Glo assay. Data are presented as mean ± SD, and P values were calculated using Student t test. * p < 0.05, ** p < 0.01, and *** p < 0.001. Figure S2. GNF-7 significantly inhibited the proliferation of Ba/F3 FLT3-ITD cells. Ba/F3 FLT3-ITD cells were treated with various concentrations of imatinib, dasatinib and GNF-7 for 48 hours. CellTiter Glo assay was applied to measure the normalized cell proliferation of these cells. Data are presented as mean ± SD, and P values were calculated using Student t test. * p < 0.05, ** p < 0.01, and *** p < 0.001. Figure S3. GNF-7 significantly induced Ba/F3 FLT3-ITD/F691L cells apoptosis. After treated with the same concentration of AC220, gilteritinib and GNF-7 for 48 hours, the apoptosis rate of Ba/F3 FLT3-ITD/F691L cells were analyzed by flow cytometry. Data are presented as mean ± SD, and P values were calculated using Student t test. * p < 0.05, ** p < 0.01, and *** p < 0.001. Figure S4. GNF-7 have potent therapy effect on FLT3-ITD harboring AML. (A-C) Primary bone marrow cells isolated from 3 diagnosed FLT3-ITD AML patients were treated with gilteritinib and GNF-7 for 48 hours and the normalized cell proliferation was measured by the CellTiter Glo assay. Data are presented as mean ± SD, and P values were calculated using Student t test. * p < 0.05, ** p < 0.01, and *** p < 0.001. Figure S5. GNF-7 inhibits the infiltration of primary blasts in PDX model. After treated with vehicle, GNF-7 and gilteritinib, three NOG mice transplanted with primary cells from AML #3 patient were randomly selected from each group and then analyzed spleen weight (A) and the content of leukemia cells in spleen were detected by flow cytometry using human CD45 antibody (B). C Spleen we [file 12935_2023_3142_MOESM1_ESM.docx]
